# Supplementary material for: Multimodal analysis unveils tumor microenvironment heterogeneity linked to immune activity and evasion
Source: iScience. 2024 Jul 15;27(8):110529. doi: 10.1016/j.isci.2024.110529 (PMC11331718; doi:10.1016/j.isci.2024.110529)
Supplement: Document S1. Figures S1‒S14 [file mmc1.pdf]

iScience, Volume *27*

Supplemental information

Multimodal analysis unveils tumor microenvironment heterogeneity linked to immune activity and evasion

Óscar Lapuente-Santana, Gregor Sturm, Joan Kant, Markus Ausserhofer, Constantin Zackl, Maria Zopoglou, Nicholas McGranahan, Dietmar Rieder, Zlatko Trajanoski, Noel Filipe da Cunha Carvalho de Miranda, Federica Eduati, and Francesca Finotello

Supplemental figures

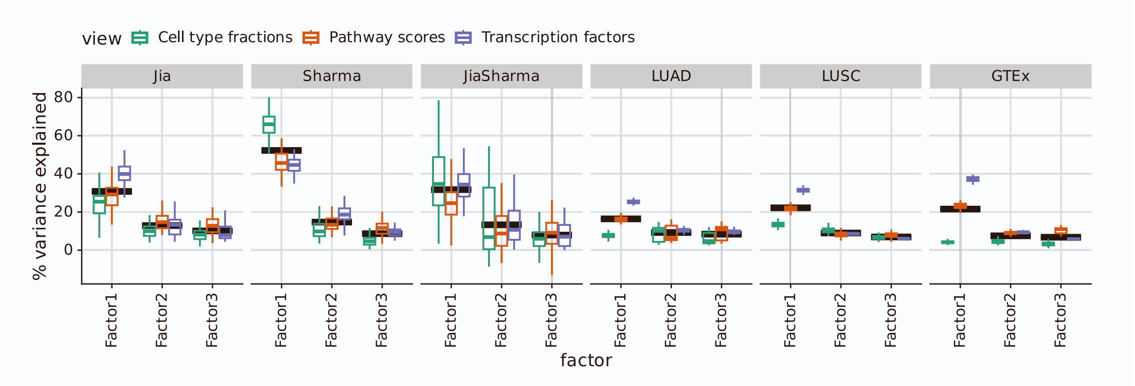

Figure S1. Explained variance per factor according to MOFA across 100 bootstrap runs, related to Figure 1. Of the boxplots, the central line denotes the median. The boxes extend to the 25 and 75 percentiles. The whiskers extend to the most extreme data point within 1.5 of the interquartile range (IQR). The black bar denotes the mean of all values.

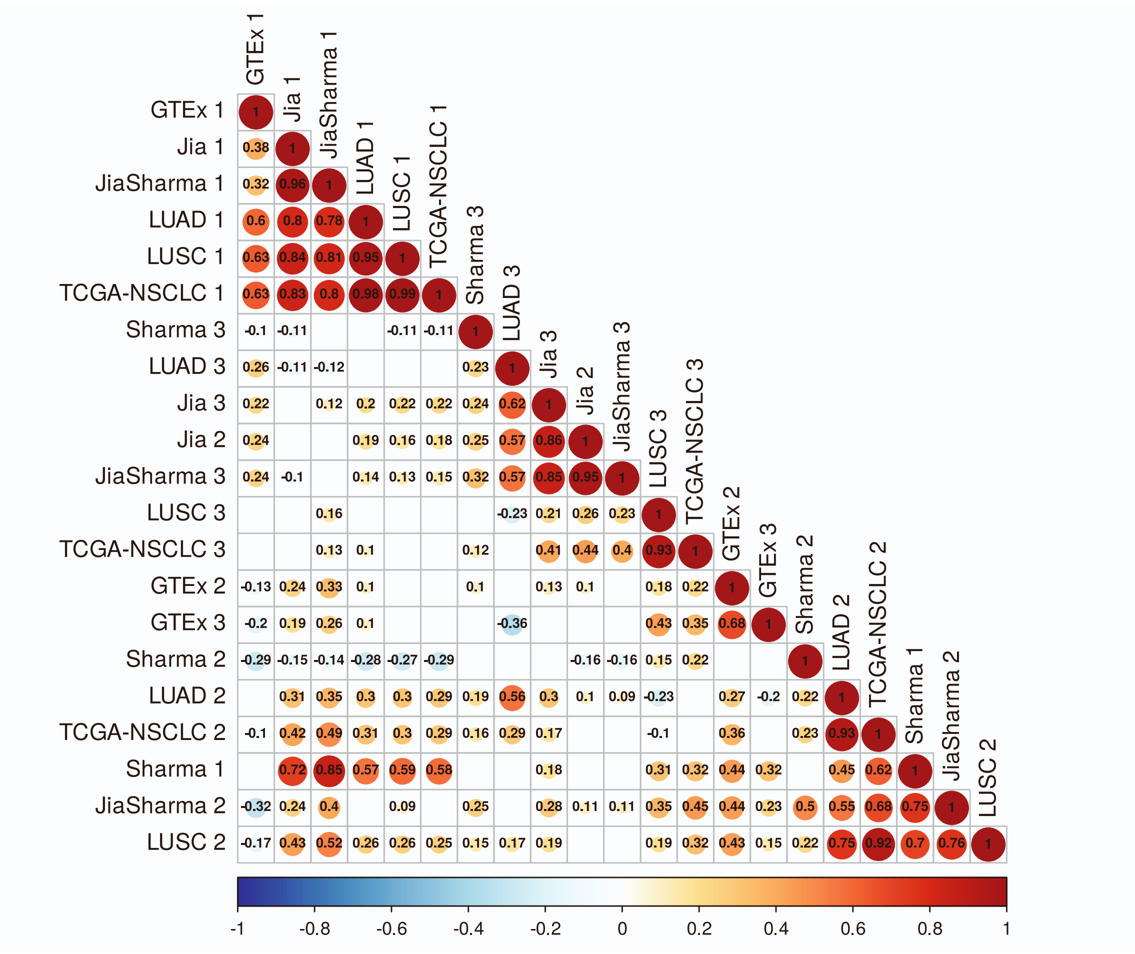

Figure S2. Correlation heatmap of F1-F3 factors for the different lung-cancer and healthy-lung datasets, related to Figure 1. Dot sizes correspond to the absolute correlation coefficient. Only dots where the FDR of the correlation is < 0.01 are shown.

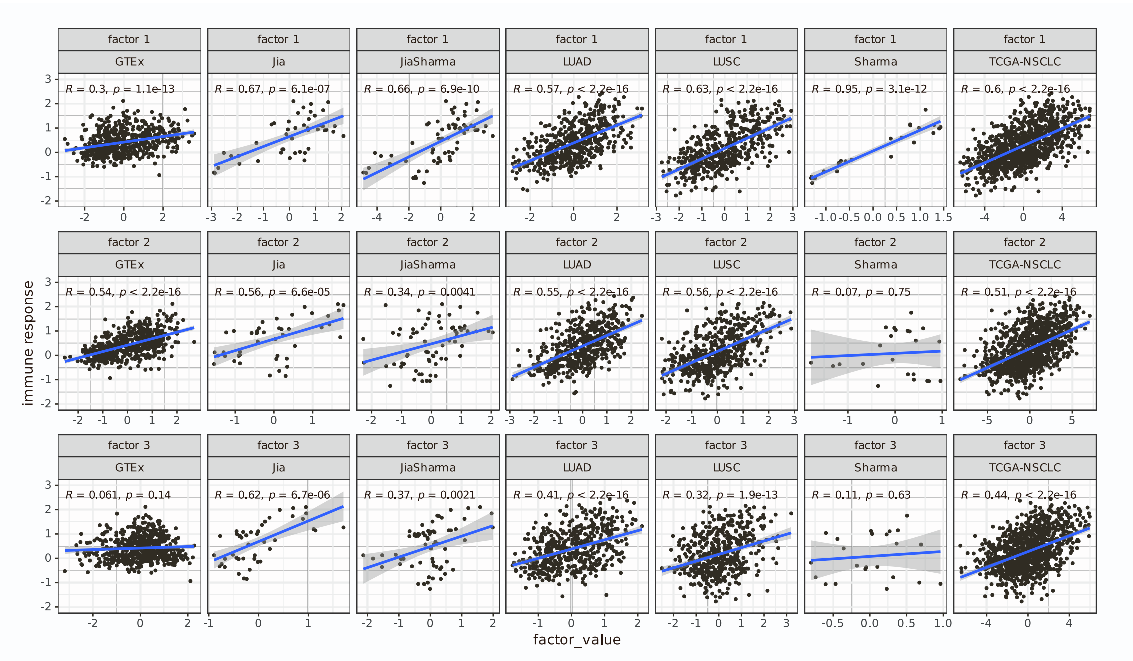

Figure S3. Correlation scatterplots of MOFA factors with the predicted immune response, related to Figure 1. Correlation of F1-F3 factors with the ensemble immune response score, derived from state-of-the-art signatures, for the datasets GTEx, Jia, JiaSharma, LUAD, LUSC, Sharma, and TCGA-NSCLC.

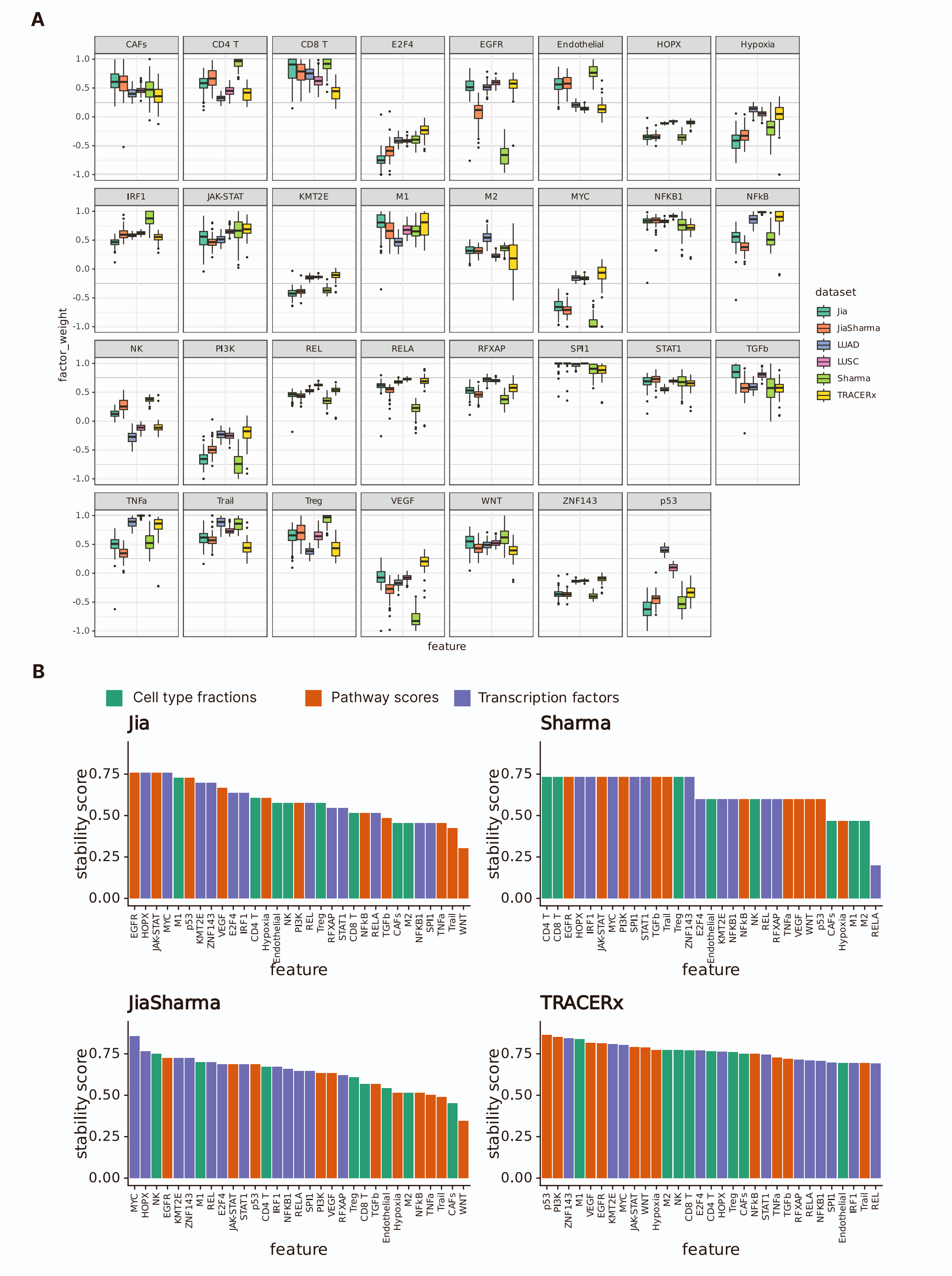

Figure S4. Feature stability across bootstrap models and tumor biopsies, related to Figure 1. (A) MOFA factor weight for top features (as highlighted in Figure 1F) across 100 bootstrap runs. The central line denotes the median. The boxes extend to the 25 and 75 percentiles. The whiskers extend to the most extreme data point within 1.5 of the interquartile range (IQR). (B) Rank-based stability score for the Jia, Sharma, JiaSharma and TRACERx datasets. A high feature stability score indicates a low intra-patient heterogeneity.

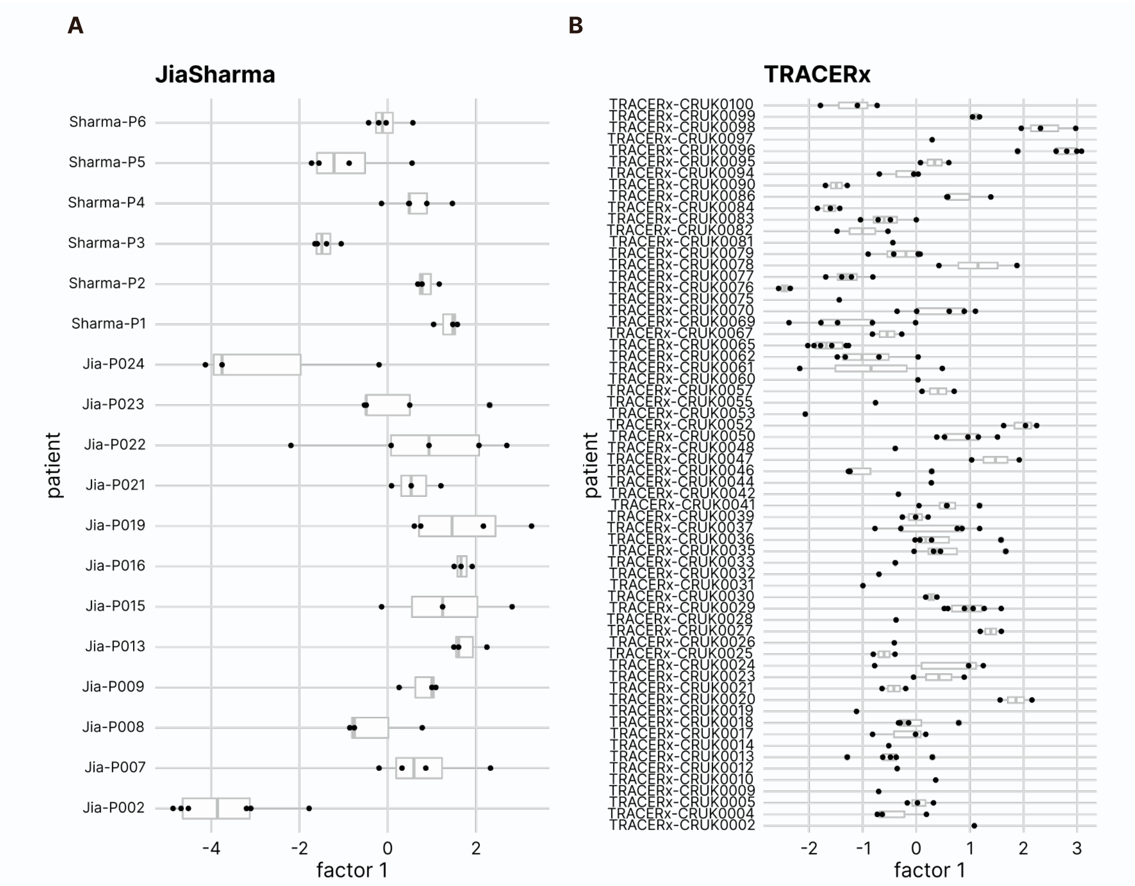

Figure S5. Intra-tumoral heterogeneity in multi-biopsy data, related to Figure 1. For each patient in the JiaSharma and TRACERx datasets, multiple biopsies were taken from different tumor locations. The figure shows the distribution of F1 weights across multiple biopsies for each patient for JiaSharma (A) and TRACERx (B). Of the boxplots, the central line denotes the median. The boxes extend to the 25 and 75 percentiles. The whiskers extend to the most extreme data point within 1.5 of the interquartile range (IQR). The black bar denotes the mean of all values.

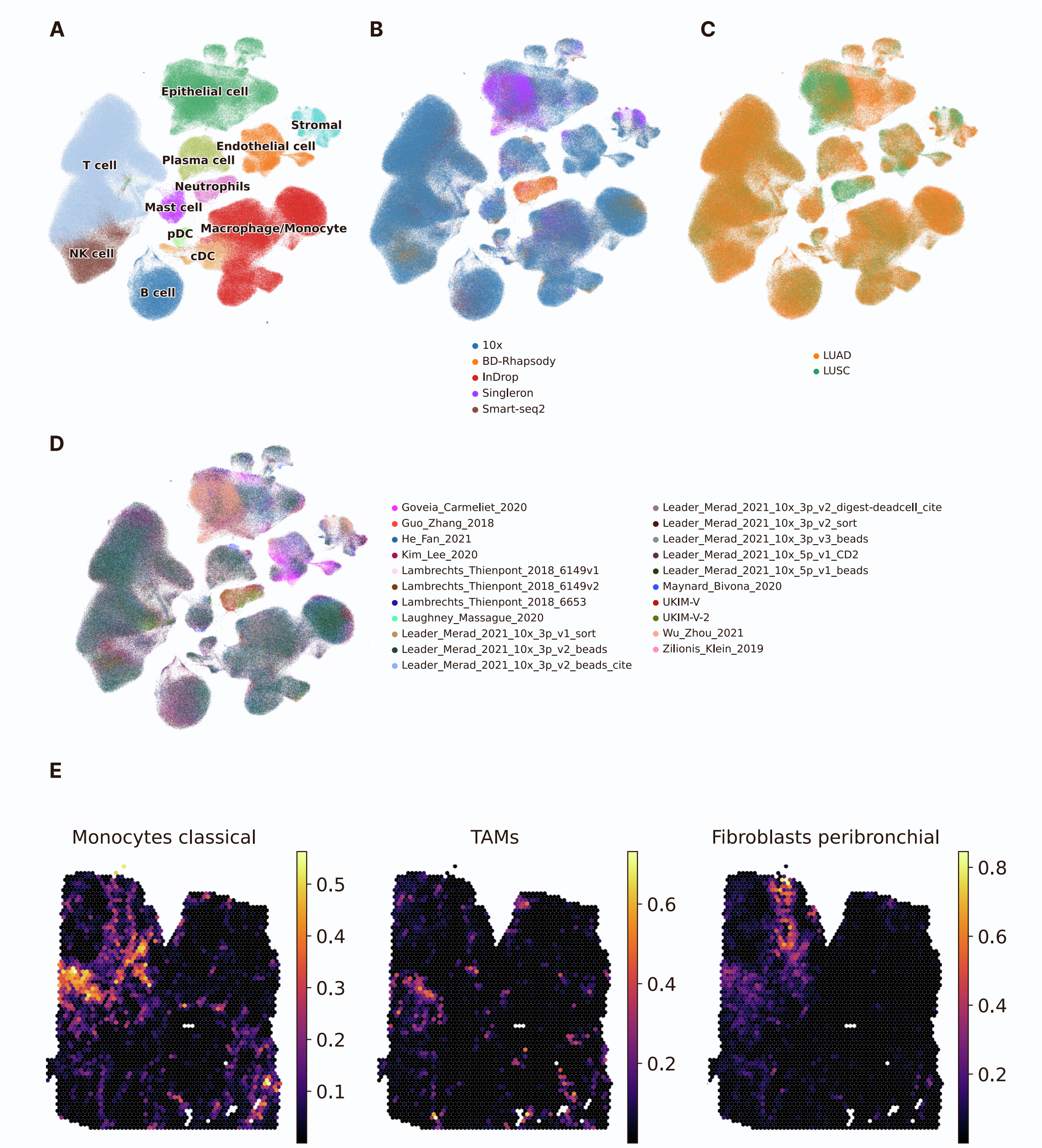

Figure S6. Covariates of the NSCLC single-cell atlas, related to Figure 2. (A-D) UMAP plot of the NSCLC single-cell atlas, colored by (A) coarse cell-type labels, (B) sequencing platform, (C) histological subtype, and (D) dataset of origin. (E) Exemplary spatial transcriptomics analysis of a lung cancer slide profiled with the 10x Visium technology (same slide as in Figure 2c). The three panels show the estimated cell-type fractions per spot for classical monocytes, tumor-associated macrophages (TAMs), and peribronchial fibroblasts, respectively.

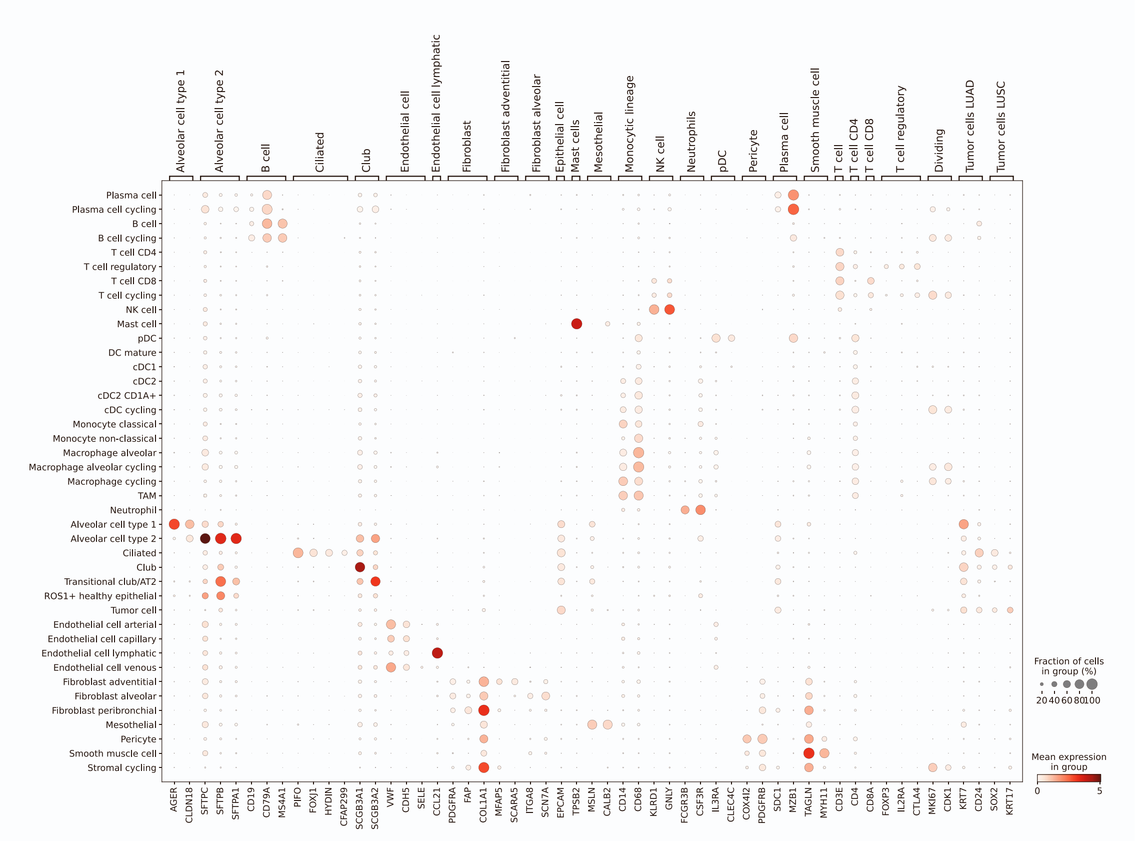

Figure S7. Cell-type markers, related to Figure 2. Dotplot showing the expression of cell-type specific marker genes across the cell-type clusters of the full single-cell dataset.

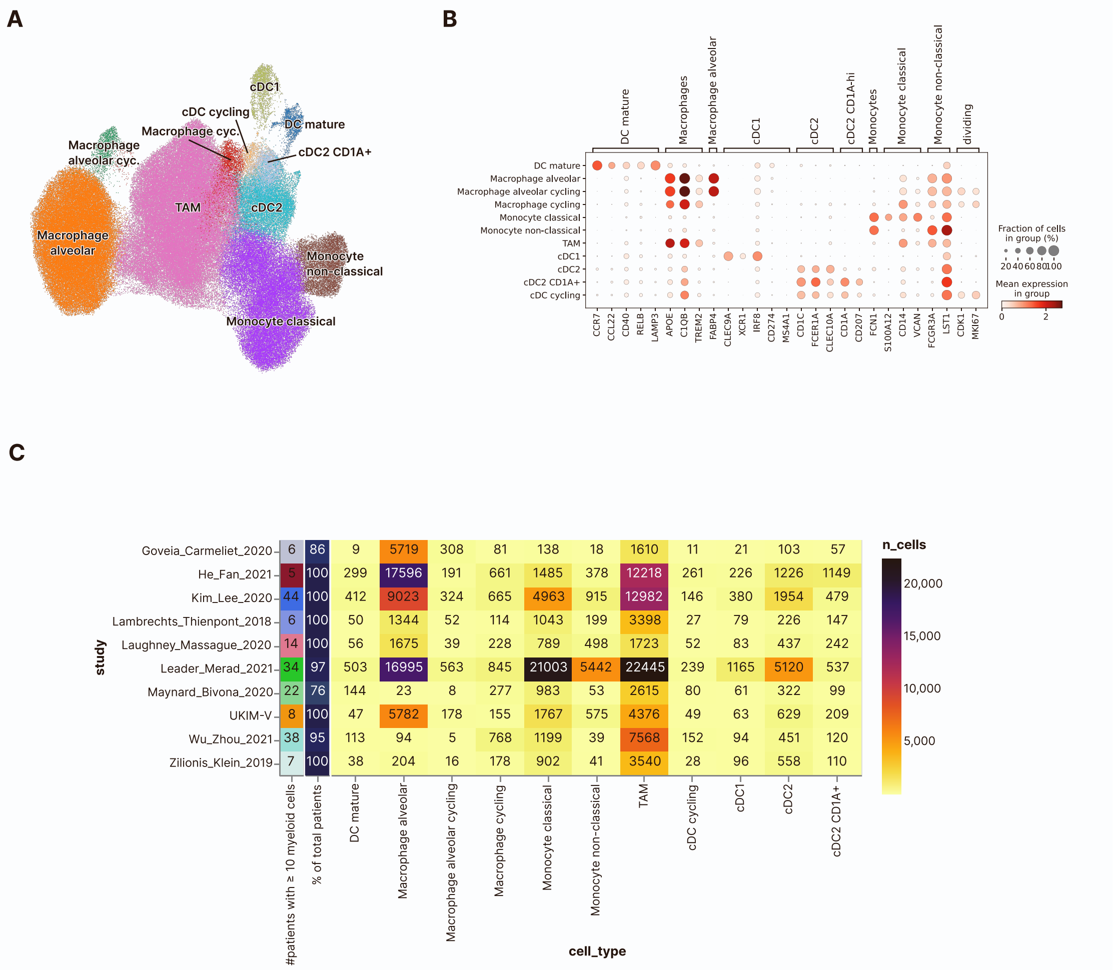

Figure S8. Analysis of the myeloid subcluster, related to Figure 2. (A) UMAP of the myeloid cluster, colored by cell-type. (B) Dotplot of marker genes characteristic for myeloid subsets. (C) Number of cells per myeloid cell-type and dataset. The first column of the matrix indicates the number of patients per dataset that have at least ten myeloid cells.

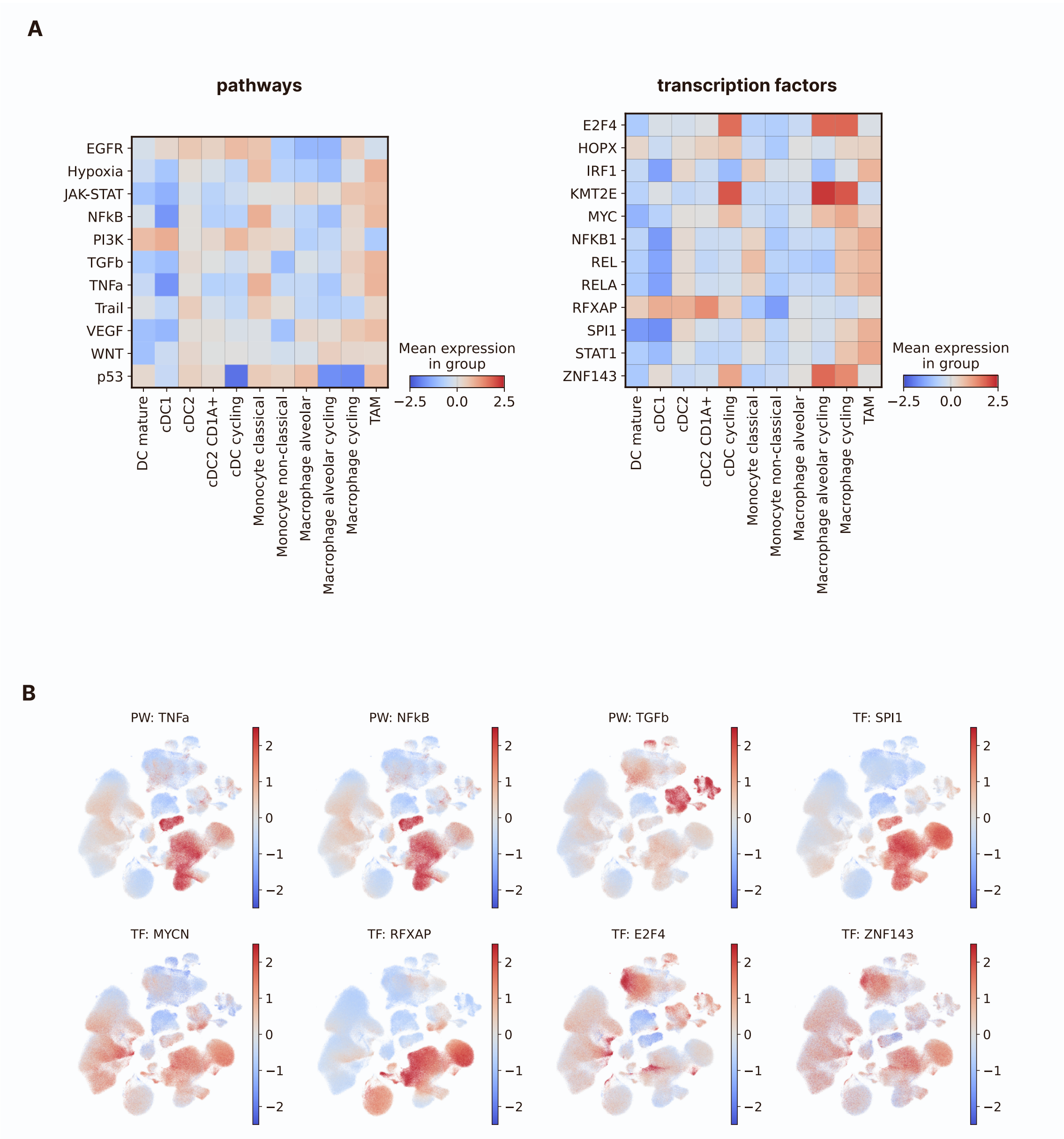

Figure S9. Pathway and transcription factor activity in myeloid cells, related to Figure 2. (A) Heatmap of average activity scores of top pathways and transcription factors (as highlighted in Figure 1F) per myeloid cell-type as determined using Progeny and Dorothea, respectively. Values are z-scores of activity scores computed for each row. (B) UMAP plots colored by the activity scores for selected pathways (PW) and transcription factors (TF).

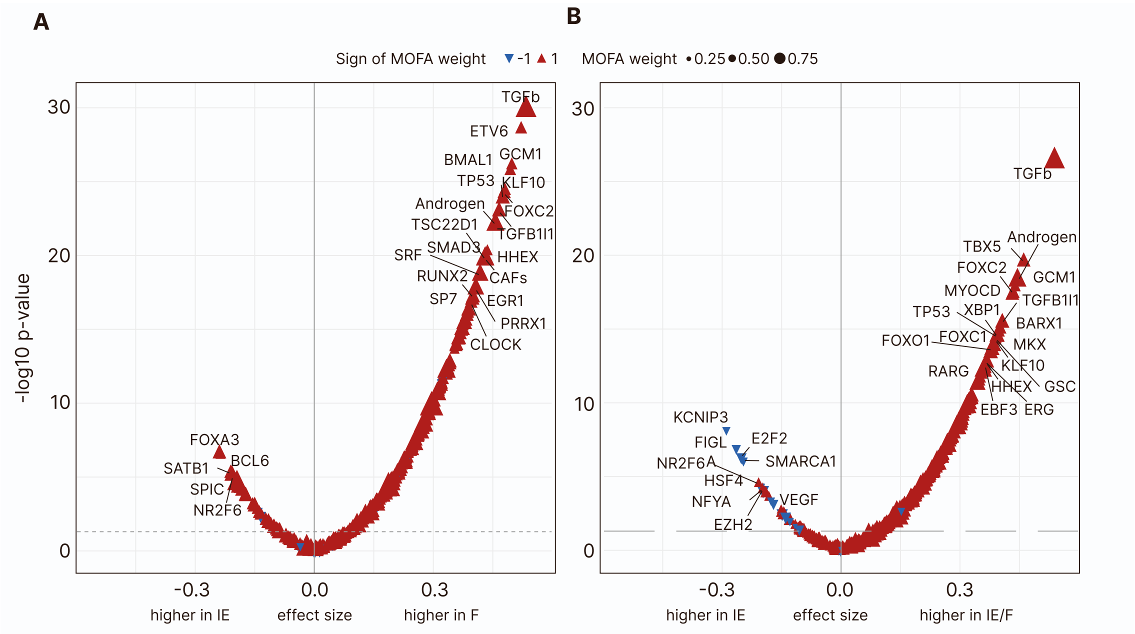

Figure S10. Comparison of features associated to immune-exclusion in patients from immune-enriched and fibrotic subtypes, related to Figure 3. (A) Immune-enriched (IE) vs immune-enriched, fibrotic (IE/F) and (B) IE vs fibrotic (F) patients (Wilcoxon rank-sum test). Each dot represents an iHet feature, where the size and color represent the corresponding weight and sign determined by MOFA, respectively.

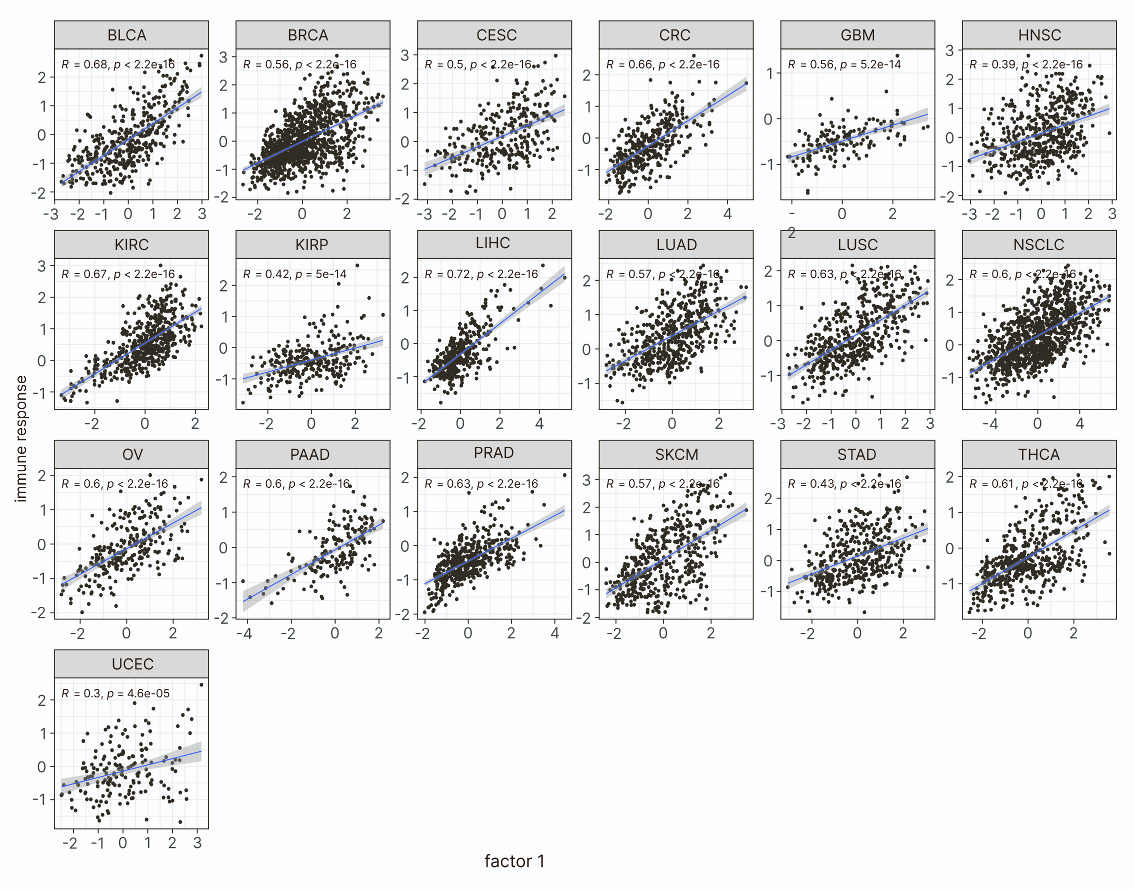

Figure S11. Correlation of iHet with immune response, related to Figure 3. Correlation scatterplots of iHet score (x-axis) with the ensemble immune response score (IR; y-axis) for each TCGA cancer type. *R* represents Pearson correlation, *p* the associated, two-tailed p-value.

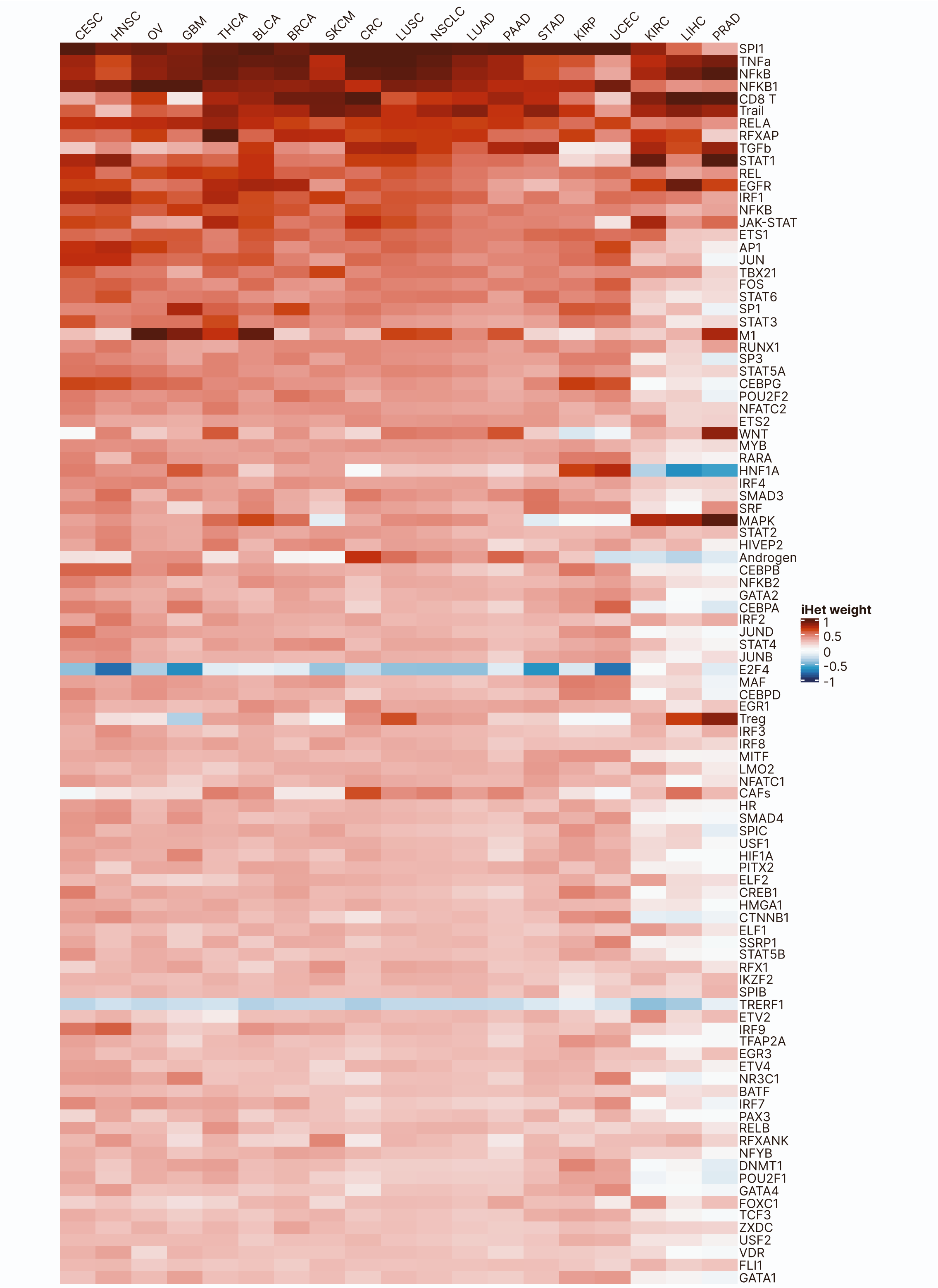

Figure S12. iHet-associated feature weights, related to Figure 3. Heatmap showing cancer-type-specific iHet-associated top 100 (ranked) feature weights. Shown are the median values computed across 100 bootstrap models. Rows (features) were sorted according to their median rank across cancer types. Columns (cancer types) were sorted based on similarity of iHet feature weight profile. Weights were scaled into the [-1,1] range in each bootstrap model.

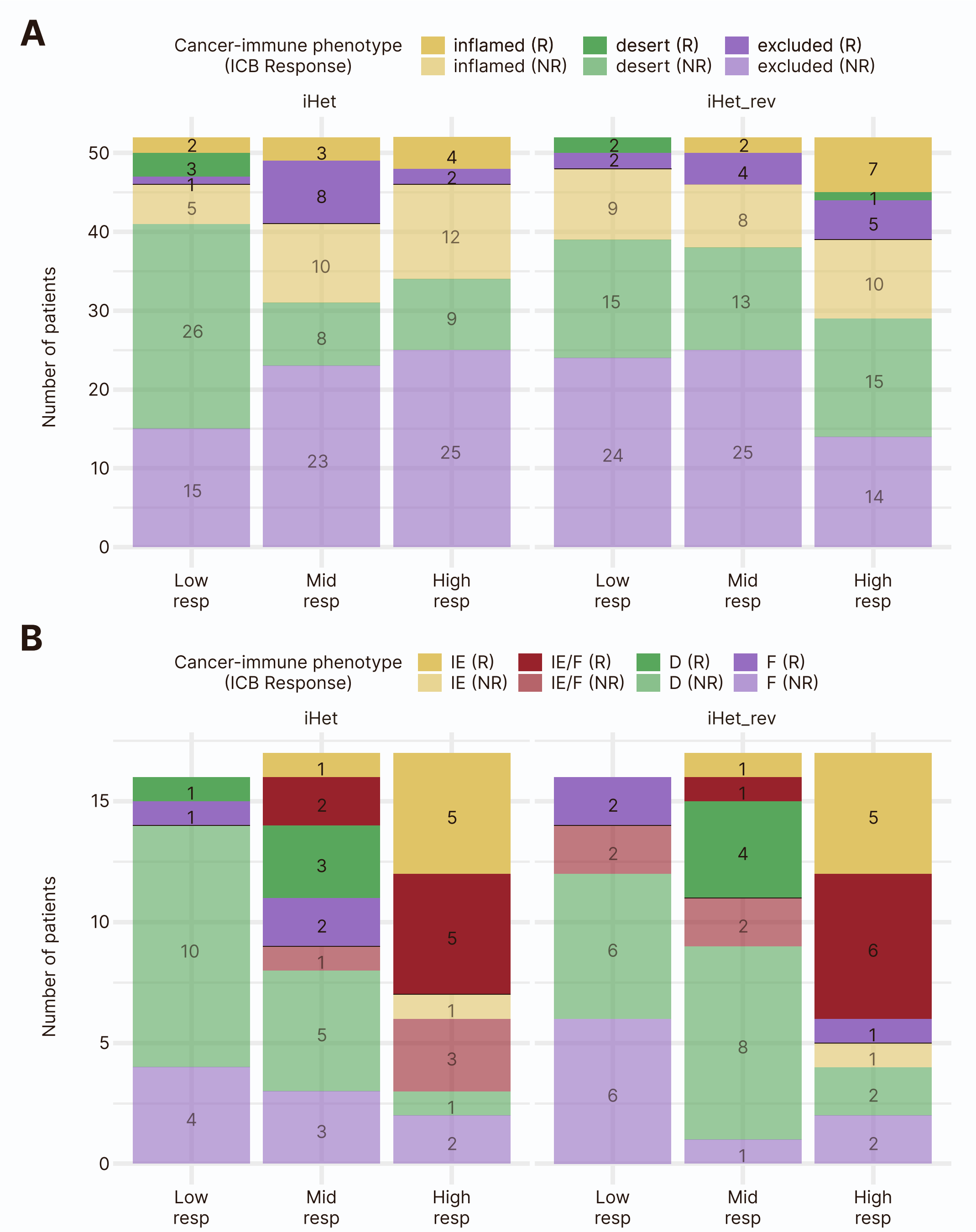

Figure S13. iHet and iHet_rev classification of patients and association with cancer-immune phenotypes, related to Figure 3. Classification of non-responding (lighter colors) and responding (darker colors) patients in three tertiles defined using iHet and iHet_rev scores for (A) the Mariathasan bladder cancer cohort, and (B) the combined Gide and Auslander melanoma cohort. Patients' are coloured according to their defined cancer-immune phenotype[1,2].

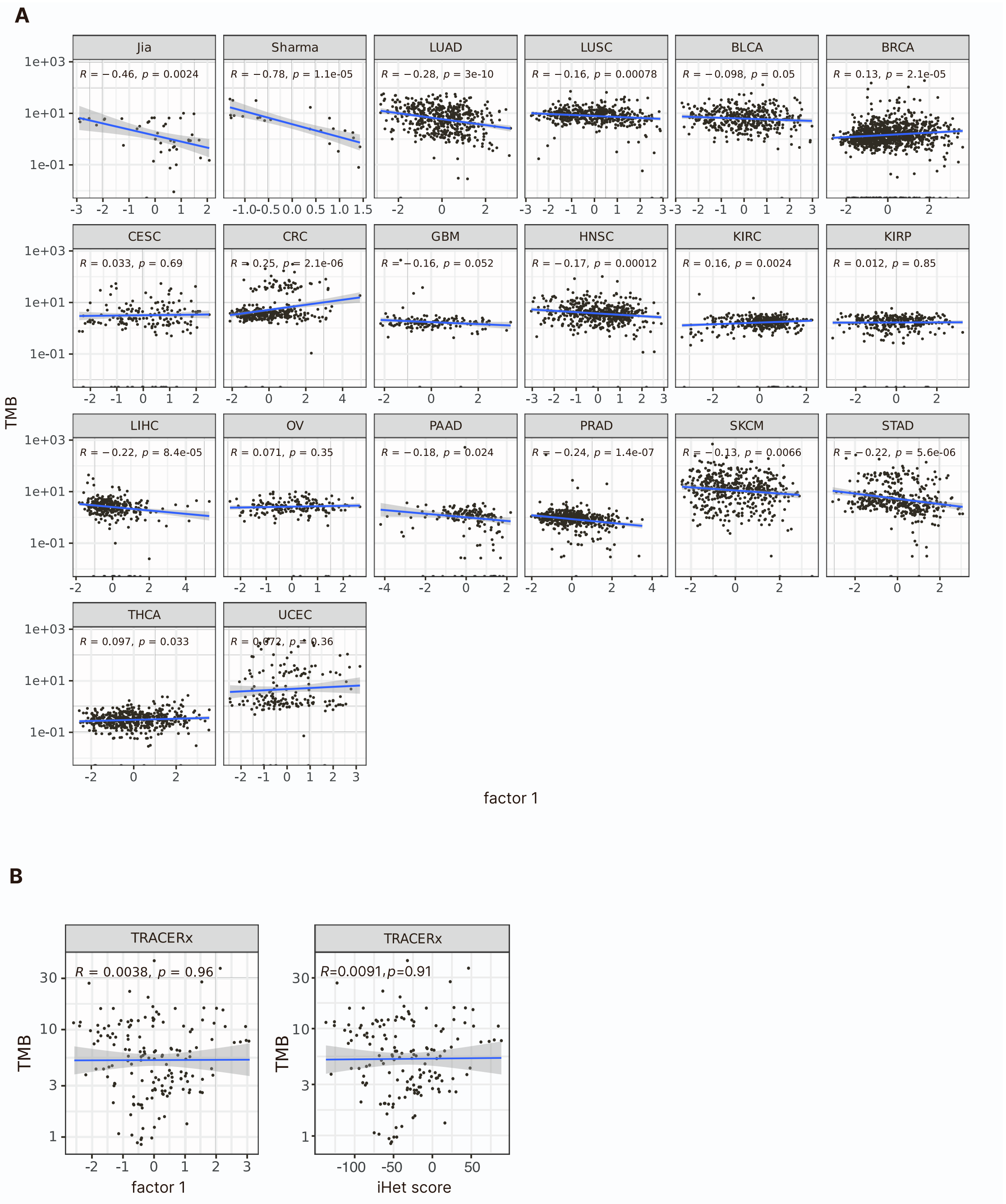

Figure S14. **Correlation of iHet with TMB, related to Figure 4.** (A) Correlation of F1 factor (estimated by MOFA), on the x-axis, with tumor mutational burden (TMB), on the y-axis, for Jia, Sharma, and all TCGA cohorts. (B) Correlation of the F1 factor (left panel) and iHet (estimated by multiplying the features scores with the F1 weights derived from the TCGA-NSCLC dataset) with TMB for the TRACERx dataset. *R* represents Pearson correlation, *p* the associated, two-tailed p-value.

Supplemental references

1. Bagaev, A., Kotlov, N., Nomie, K., Svekolkin, V., Gafurov, A., Isaeva, O., Osokin, N., Kozlov, I., Frenkel, F., Gancharova, O., et al. (2021). Conserved pan-cancer microenvironment subtypes predict response to immunotherapy. Cancer Cell *39*, 845–865.e7.

2. Mariathasan, S., Turley, S.J., Nickles, D., Castiglioni, A., Yuen, K., Wang, Y., Kadel, E.E., III, Koeppen, H., Astarita, J.L., Cubas, R., et al. (2018). TGFβ attenuates tumour response to PD-L1 blockade by contributing to exclusion of T cells. Nature *554*, 544–548.
